# Supplementary material for: Single-cell multi-omics identifies chronic inflammation as a driver of TP53-mutant leukemic evolution
Source: Nat Genet. 2023 Sep 4;55(9):1531–41. doi: 10.1038/s41588-023-01480-1 (PMC10484789; doi:10.1038/s41588-023-01480-1)
Supplement: Supplementary file 1 — Reporting Summary [file 41588_2023_1480_MOESM1_ESM.pdf]

nature portfolio

Corresponding author(s): Adam J Mead, Iléana Antony-Debré, Alba Rodriguez-Meira

Last updated by author(s): Jun 19, 2023

Reporting Summary

Nature Portfolio wishes to improve the reproducibility of the work that we publish. This form provides structure for consistency and transparency in reporting. For further information on Nature Portfolio policies, see our Editorial Policies and the Editorial Policy Checklist.

Statistics

For all statistical analyses, confirm that the following items are present in the figure legend, table legend, main text, or Methods section.

n/a	Confirmed	
☐	☒	The exact sample size (*n*) for each experimental group/condition, given as a discrete number and unit of measurement
☐	☒	A statement on whether measurements were taken from distinct samples or whether the same sample was measured repeatedly
☐	☒	The statistical test(s) used AND whether they are one- or two-sided<br>*Only common tests should be described solely by name; describe more complex techniques in the Methods section.*
☐	☒	A description of all covariates tested
☐	☒	A description of any assumptions or corrections, such as tests of normality and adjustment for multiple comparisons
☐	☒	A full description of the statistical parameters including central tendency (e.g. means) or other basic estimates (e.g. regression coefficient) AND variation (e.g. standard deviation) or associated estimates of uncertainty (e.g. confidence intervals)
☐	☒	For null hypothesis testing, the test statistic (e.g. *F*, *t*, *r*) with confidence intervals, effect sizes, degrees of freedom and *P* value noted<br>*Give P values as exact values whenever suitable.*
☐	☒	For Bayesian analysis, information on the choice of priors and Markov chain Monte Carlo settings
☒	☐	For hierarchical and complex designs, identification of the appropriate level for tests and full reporting of outcomes
☒	☐	Estimates of effect sizes (e.g. Cohen's *d*, Pearson's *r*), indicating how they were calculated

Our web collection on statistics for biologists contains articles on many of the points above.

Software and code

Policy information about availability of computer code

Data collection

- Single cell RNA-seq and genotyping libraries were generated using 3'-TARGET-seq (Rodriguez-Meira et al, 2019) at the University of Oxford. Libraries were sequenced in paired-end format on an Illumina HiSeq2000, HiSeq4000 or NextSeq. FASTQ files were generated using bcl2fastq (version 2.20) .
- Targeted myeloid-panel sequencing libraries were generated from bulk genomic DNA using a TruSeq Custom Amplicon panel (Illumina) or a Haloplex Target Enrichment System (Agilent technologies), and were sequenced on a MiSeq (Illumina) instrument.
- SNP-Array data was generated through hybridization of bulk genomic DNA to an Illumina Infinium OmniExpress v1.3 BeadChips Array and SNP-CGH CytoScan HD Array
- Flow cytometry data was collected using BD FACS Diva Software (version 8.0.2).
- Cells were sorted on an Influx (BD Biosciences), a BD Fusion I and BD Fusion II instruments (Becton Dickinson), a SH800S or MA900 (SONY) cell sorters.
- Images for cell morphology were obtained using an AxioPhot microscope (Zeiss).
- Images for M-FISH were obtained with an Olympus BX-51 epifluorescence microscope

Data collection methods are fully described in the manuscript.

Data analysis

- Bioinformatic analysis were performed with CentOS Linux 7, R (v4.0.5 and v3.6.1), Python 3.9.13.
Linux packages: bcl2fastq v2.20, Bedtools v2.27.1, BWA v0.7.17, FeatureCounts v1.4.5-p1, GATK v4.1.2.0, Picard v2.3.0, Samtools v.19, SCITE v2016b31, STAR v2.6.1d and v2.4.2a, TrimGalore v0.4.1.
R packages: bigmemory v4.5.36, Biobase v2.50.0, BiocGenerics v0.44.0, cccd v1.5, circlize v0.4.12, clusterProfiler v3.18.1, ComplexHeatmap v2.11.1, data.table v1.14.0, destiny v3.9.0, diffusionMap v1.2.0, DNAcopy v1.60.0, fgsea v1.16.0, GeneOverlap v1.26.0, GenomicRanges v1.42.0, GenomeInfoDb v1.34.9, GISTIC2 v2.023, ggplot2 v3.4.2, ggpubr v0.4.0, glmnet v4.1-1, gridExtra v2.3, igraph v1.2.6, IRanges v2.24.1,

irlba v2.3.3, inferCNV v1.0.4, limma v3.46.0, LinkedMatrix v1.4.0, Matrix v1.5-1, MatrixGenerics v1.2.1, matrixStats v0.58.0, Mocha WDL pipeline v2021-01-20 (https://software.broadinstitute.org/software/mocha/mocha.20210120.wdl); pbapply v1.4-3, pheatmap v1.0.12, RANN v2.6.1, RColorBrewer v1.1-2, Rcpp v1.0.7, SHAPEIT v4.1.3.
reshape2 v1.4.4, reticulate v1.22, Rtsne v0.15, S4Vectors v0.28.1, SingCellaR v1.2.0/v1.2.1, SingleCellExperiment v1.12.0, statmod v1.4.36, SummarizedExperiment v1.20.0, survival v3.5-5, survminer v0.4.9, threejs v0.3.3, umap v0.2.7.0
Python packages: Velocyto v0.17.13
GSEA software version 4.0.3, http://software.broadinstitute.org/gsea
- Targeted myeloid-panel sequencing data : SOPHiA DDM® (Sophia Genetics) and an in-house software GRIO-Dx®.
- SNP Array data : Chromosome Analysis Suite (Affymetrix) v4.1
- M-FISH : Leica Cytovision software version 7.3.1
- Flow cytometry : Kaluza (version 2.1, Beckman Coulter) or FlowJo (version 10.1, BD Biosciences) softwares
- Statistical analysis : GraphPad Prism software (version 7 or later)
- Custom codes are available on https://github.com/albarmeira/p53-transformation

All methods for data analyses are fully described in the methods section of the manuscript.

For manuscripts utilizing custom algorithms or software that are central to the research but not yet described in published literature, software must be made available to editors and reviewers. We strongly encourage code deposition in a community repository (e.g. GitHub). See the Nature Portfolio guidelines for submitting code & software for further information.

Data

Policy information about availability of data

All manuscripts must include a data availability statement. This statement should provide the following information, where applicable:

- Accession codes, unique identifiers, or web links for publicly available datasets
- A description of any restrictions on data availability
- For clinical datasets or third party data, please ensure that the statement adheres to our policy

Database/datasets used in this study:
- Database: COSMIC (Catalogue Of Somatic Mutations In Cancer, https://cancer.sanger.ac.uk/cosmic) , Pecan Portal, genome version GRCh37 (hg19), National Cancer Institute (NIH) Genomic Data Commons, cBio Cancer Genomics Portal
- Datasets from the following papers : Papaemmanuil et al, N Engl J Med, 2016; Coombs et al, Cell Stem Cell, 2017; Desai et al, Nat Med, 2016 ; Young et al, Nat Commun, 2016; Psaila et al, Mol Cell, 2020; van Galen et al, Cell, 2019; Fisher et al, Oncogene, 2017; Granja et al, Nature Biotechnology, 2019 ; Ng et al, Nature, 2016.
Two publicly available AML cohorts with genetic mutation and RNA-sequencing data: BeatAML (Tyner et al, Nature, 2018) and The Cancer Genome Atlas (TCGA) (Ley et al, N Engl J Med, 2013). Subsets of single-cell genotyping and RNA-sequencing data were part of a previously published study (Rodriguez-Meira et al, Mol Cell, 2019).
Data generated in the study : Single-cell genotyping and RNA-sequencing data generated from this study are publicly available in SRA and GEO with accession numbers PRJNA930152 and GSE226340, respectively. The dataset generated in this paper is also available as an interactive vignette https://wenweixiong.shinyapps.io/TP53_MPN_AML_Single_Cell_Atlas/.

Research involving human participants, their data, or biological material

Policy information about studies with human participants or human data. See also policy information about sex, gender (identity/presentation), and sexual orientation and race, ethnicity and racism.

Reporting on sex and gender	Sex and gender were not take into account to select the population of interest.
Reporting on race, ethnicity, or other socially relevant groupings	Socially relevant groupings were not take into account to select the population of interest.
Population characteristics	Patients samples were selected based on their pathology (patients with myeloproliferative neoplasms or acute myeloid leukemia secondary to a myelopoliferative neoplasm, or TP53-sAML) and on TP53 status (mutated or wild-type). Age-matched healthy donors were also used for the study.
Recruitment	Samples were collected as part of patients' routine clinical care through previously established research study approvals as detailed below. Patients and normal donors provided written informed consent in accordance with the Declaration of Helsinki for sample collection and use in research.
Ethics oversight	Samples were obtained from:<br>- Gustave Roussy (Villejuif, France) and Dijon Hospital (Dijon, France) with the agreement from the Inserm Institutional Review Board Ethical Committee (project C19-73, agreement 21-794, CODECOH n°DC-2020-4324).<br>- from the INForMeD Study (REC: 199833, 26 July 2016, University of Oxford).<br>Patients and healthy donors provided written informed consent in accordance with the Declaration of Helsinki for sample collection and use in research.

Note that full information on the approval of the study protocol must also be provided in the manuscript.

Field-specific reporting

Please select the one below that is the best fit for your research. If you are not sure, read the appropriate sections before making your selection.

☒ Life sciences ☐ Behavioural & social sciences ☐ Ecological, evolutionary & environmental sciences

For a reference copy of the document with all sections, see nature.com/documents/nr-reporting-summary-flat.pdf

Life sciences study design

All studies must disclose on these points even when the disclosure is negative.

Sample size	Sample size was determined based on similar studies in the field and availability of samples/numbers of cells per sample.
Data exclusions	Data which didn't meet quality control parameters (as detailed in Methods section) were excluded from the analysis.
Replication	In vitro and in vivo experiments were repeated to reach 3 biological replicates in at least 2 independent experiments. Attempts at replication were successful. Details on numbers of replicates are provided in the relevant legend and/or methods section.
Randomization	Patient samples were separated according to their diagnosis and TP53 mutational status, randomization was not appropriate. Mice were allocated randomly to control or treated groups.
Blinding	Blinding was not relevant for single cell data, as the information on mutational status was required for analysis. For mouse experiments, blinding was performed for analysis of FACS data with an anonymized identification number for each mouse. For M-FISH karyotype assessment, the operator was blinded to the genotype and treatment group.

Reporting for specific materials, systems and methods

We require information from authors about some types of materials, experimental systems and methods used in many studies. Here, indicate whether each material, system or method listed is relevant to your study. If you are not sure if a list item applies to your research, read the appropriate section before selecting a response.

Materials & experimental systems

n/a	Involved in the study	
☐	☒	Antibodies
☒	☐	Eukaryotic cell lines
☒	☐	Palaeontology and archaeology
☐	☒	Animals and other organisms
☒	☐	Clinical data
☒	☐	Dual use research of concern
☒	☐	Plants

Methods

n/a	Involved in the study	
☒	☐	ChIP-seq
☐	☒	Flow cytometry
☒	☐	MRI-based neuroimaging

Antibodies

Antibodies used

- Antibodies used for human HSPC sorting.
CD8-FITC (Lineage), BioLegend, Clone: RPA-T8, Cat#: 301006, 1/100
CD20-FITC (Lineage), BioLegend, Clone: 2H7, Cat#: 302304 , 1/150
CD66b-FITC (Lineage), BioLegend, Clone: G10F5, Cat#: 305104, 1/15
CD10-FITC (Lineage), BioLegend, Clone: HI10a, Cat#: 312208, 1/30
CD127-FITC (Lineage), eBioscience, Clone eBioRDR5, Cat#: 11-1278-42, 1/30
Human Hematopoietic Lineage Cocktail – FITC (Lineage), eBioscience, NA , Cat# 22-7778-72, 1/15
CD123-PECy7, BioLegend, Clone: 6H6, Cat#: 306010, 1/60
CD38-PETxRed, Invitrogen, Clone: HIT2, Cat#: MHCD3817, 1/21.5
CD90-BV421, BioLegend, Clone: 5E10, Cat#: 328122, 1/30
CD45RA-PE, eBioscience, Clone: HI100, Cat#: 12-0458-41, 1/150
CD34-APC-eF780, eBioscience, Clone: 4H11, Cat#: 47-0349-42, 1/150
CD34-PerCP/Cy5.5, BioLegend, Clone: 562, Cat# 343611, 1/100
CD90-PE, BioLegend, Clone: 5E10, Cat# 328109, 1/25
CD45RA-FITC, Invitrogen, Clone: MEM56, Cat# MHCD45RA01, 1/150
CD2-PE/Cy5, BioLegend, Clone: RPA-2.10, Cat# 300209, 1/300
CD3-PE/Cy5, BioLegend, Clone: HIT3a, Cat# 300310, 1/300
CD4-PE/Cy5 , BioLegend, Clone: RPA-T4, Cat# 300510, 1/160
CD8-PE/Cy5, BioLegend, Clone: RPA-T8, Cat# 301010, 1/300
CD10-PE/Cy5, BioLegend, Clone: HI10a, Cat# 312206, 1/80
CD11b-PE/Cy5, BioLegend, Clone: ICRF44, Cat# 301308, 1/160
CD14-PE/Cy5, Invitrogen, Clone: 61D3, Cat# 15-0149-41, 1/160

CD19-PE/Cy5, BioLegend, Clone: HIB19, Cat# AB_314240, 1/300
CD20-PE/Cy5, BioLegend, Clone: 2H7, Cat# AB_314256, 1/200
CD56-PE/Cy5, BD Biosciences, Clone: B159, Cat# 555517, 1/80
CD235a,b-PE/Cy5, BioLegend, Clone: HIR2, Cat# 306606, 1/300
CD117-APC, BD Pharmigen, 104D2, Cat# 333233, 1/30
- Antibodies used for PDX model flow cytometry readout and sorting
CD2-APC-F750, Biolegend, RPA-2.10, 300225, 1/160
CD3-APC-F750, Biolegend, SK7, 344839, 1/160
CD7-APC-F750, Biolegend, CD7-6B7, 343121, 1/160
CD11b-APC-F750, Biolegend, ICRF44 301351, 1/160
CD14-APC-F750, Biolegend, 63D3, 367120, 1/160
CD19-APC-F750, Biolegend, SJ25C1, 363029, 1/160
CD20-APC-F750, Biolegend, 2H7, 302357, 1/160
CD56-APC-F750, Biolegend, 5.1H11, 362553, 1/160
CD235a-APC-F750, Biolegend, HIR2, 306622, 1/160
CD90-PECy7, Biolegend, 5E10, 328124, 1/20
CD34-APC, Biolegend, 581, 343510, 1/100
CD34-PECy7, Beckman Coulter, 581, A21691, 1/100 or 1/50
CD38-BV421, Biolegend, HIT2, 303526, 1/20
CD123-PE, Biolegend, 6H6, 306005, 1/40
CD45RA-BB515, BD Biosciences, HI100, 564552, 1/40
mCD45-BV605, BD Biosciences, 30-F11, 563053, 1/40
mCD45-APC, BD Biosciences, 30-F11 , 559864, 1/100
hCD45-BV786, BD Biosciences, HI30, 563716, 1/40 or 1/50
hCD45-PE, BD Biosciences, HI30, 555483, 1/100
CD41-APC-F750, Biolegend, HIP8, 303749, 1/160
CD235a-PE, BD Biosciences, GA-R2, 555570, 1/50
CD71-BV605, BD Biosciences, M-A712, 743306, 1/50
CD36-APC, BD Biosciences, CB38, 550956, 1/50
CD123-FITC, Biolegend, 6H6, 306013, 1/50
CD117-BV711, Biolegend, 104D2, 313229, 1/50
mCD45-PerCP-Cy5.5, Biolegend, F11 , 103131, 1/50
- Antibodies used for human in vitro differentiation flow cytometry readout.
CD34-PECy7, Beckman Coulter, 581, A21691, 1/100
CD41-AP, BD Biosciences, HIP8, 559777, 1/100
CD42-PE, BD Biosciences, ALMA.16, 558819, 1/100
CD71-BV605, BD Biosciences, M-A712, 743306, 1/100
CD14-AF700, Biolegend, 63D3, 367114, 1/100
CD15-AF700, Biolegend, HI98, 301920, 1/100
CD11b-AF700, Biolegend, ICRF44, 301356, 1/100
CD235a-APC-F750, Biolegend, HIR2, 306622, 1/100
CD34-APC-e780, eBiosciences, 4HI1, 47-0349-42, 1/150
CD41a-APC, eBioscience, HIP8, 17-0419-42, 1/37.5
CD42b-PE, Biolegend, HIP1, 303906, 1/60
CD71-AF700, BD Pharmigen, M-A712, 563769, 1/30
CD117-BV711, Biolegend, 104D2, 313230, 1/60
CD33-PECy7, Biolegend, P67.6, 366618, 1/150
CD235a-BV421, BD Horizon, GA-R2, 562938 , 1/300
CD14-FITC, eBiosciences, 61D3, 11-0149-42, 1/75
CD11b-FITC, eBioSciences, ICRF44, 11-0118-42, 1/75
- Antibodies used for mouse peripheral blood and bone marrow flow cytometry readouts.
CD45.1-BV605, BioLegend, A20, 110738, 1/100
CD45.2 AF700, BioLegend, 104, 109822, 1/100 BM, 1/400 PB
Mac1 PE-Cy7, BioLegend, M1/70, 101215, 1/1600
CD19 eF450, eBioscience, eBio1D3, 48-0193-82, 1/150
CD4 BV650, BioLegend, RM4-5, 100546, 1/200
CD8a APCeF780, eBioscience, 53-6.7, 47-0081-82, 1/400
NK1.1-PE-Cy5, BioLegend, PK136, 108716, 1/400
Ly6G FITC, BioLegend, 1A8, 127606, 1/100
CD11b PE, BioLegend, M1/70, 101208, 1/100
CD3 Pacific Blue, BioLegend, 17A2, 100214, 1/100
B220 APC-Cy7 , BioLegend, RA3-6B2, 103224, 1/100
NK1.1 APC-Cy7, BioLegend, PK136, 108724, 1/100
CD45.1 PE-Cy7, BioLegend, A20, 110730, 1/100
CD45.2 APC, BioLegend, 104, 109814, 1/100
CD4 PE-Cy5, BioLegend, RM4-5, 100514, 1/400
B220 PE-Cy5, BioLegend, RA3-6B2, 103210, 1/200
Gr1 PE-Cy5, BioLegend, RB6-8C5, 108410, 1/800
CD5 PE-Cy5, BioLegend, 53-7.3, 100610, 1/800
Mac1 PE-Cy5, BioLegend, M1/70, 101210, 1/800
CD8a PE-Cy5, BioLegend, 53-6.7, 100710, 1/1200
cKIT APC-eF780, eBioscience, 2B8, 47-1171-82, 1/400
Sca1 BV05, BioLegend, D7, 108133, 1/200
CD150 PE-Cy7, BioLegend, TC15-12F12.2, 115914, 1/400
CD48 APC, BioLegend, HM48-1, 103412, 1/600
CD45.1 FITC, BioLegend, A20, 110706, 1/400

Sca1 PE-Cy7, BioLegend, E13-161.7, 122514 , 1/600
Ter119 PE-Cy5, BioLegend, TER-119, 116210, 1/400
Ter119, BioLegend, TER119, 116202, 5 µL / 25.106 cells
B220, BioLegend, RA3-6B2, 103202, 5 µL / 25.106 cells
Gr1, BioLegend, RB6-8C5, 108402, 6.25 µL / 25.106 cells
CD4, BioLegend, GK1.5, 100402, 1.25 µL / 25.106 cells
CD8, BioLegend, 53-6.7, 100702, 1.25 µL / 25.106 cells
CD11b, BioLegend, M1/70, 101202, 2.5 µL / 25.106 cells
B220 APC, BioLegend, RA3-6B2, 103212, 1/100
Ter119 APC, BioLegend, TER119, 116212, 1/100
Gr1 APC, BioLegend, RB6-8C5, 108412, 1/100
CD3 APC, BioLegend, 17A2, 100236, 1/100
CD11b APC, BioLegend, R1/70, 101212, 1/100
c-Kit PerCP-Cy5.5, BioLegend, 2B8, 105824, 1/50
Sca1 PE-Cy7, BioLegend, D7, 108114 , 1/50
CD150 PE, BioLegend, TC15-12F12.2 , 115904, 1/50
FcGR APC-Cy7, BioLegend, 93, 101327, 1/50
CD41 AF700, BioLegend, MWReg30, 133926, 1/50
CD105 BV786, BDHorizon, MJ7/18, 564746, 1/50
CD48 BV711, BioLegend, HM48-1, 103441, 1/50
CD45.1 BUV 395, BDHorizon, A20, 565212, 1/25
CD45.2 BUV737, BDHorizon, 104, 612778, 1/25
AnnexinV FITC, BioLegend, NA, 640906, 1/100
Ki67 FITC, BioLegend, 16A8, 652410, 1/25

Validation

Human and mouse antibodies were already validated, titrated and referenced in peer-reviewed publications, as described on the suppliers' websites (Biolegend, eBiosciences, BD horizon, BD Biosciences, BD Pharmingen, Beckman Coulter). Combination of antibodies for human hematopoietic stem cells and progenitors have been already tested in our previous publication (Rodriguez-Meira et al, Mol Cell, 2019).

Animals and other research organisms

Policy information about studies involving animals; ARRIVE guidelines recommended for reporting animal research, and Sex and Gender in Research

Laboratory animals

Mouse housing was carried out in individually ventilated cages (19-24°C, humidity 40-65%, 12/12 light dark cycle). Enrichment was done with nesting and bedding material. Mice were fed on standard croquettes, and supplemented with nutritionally complete gel diet after irradiation and in case of weight loss. Mice were maintained on a specific and opportunistic pathogen free health status.

- NOD.CB17-Prkdcscid IL2rgtm1/Bcgen mice (B-NDG, Envigo), female, 8 weeks-old (PDX experiments)
- C57/BL6 wild type mice CD45.1 (11-17 weeks old) or CD45.2 (6-8 weeks old), male and female (for chimera experiments with poly(I:C) and LPS challenge)
- C57/BL6 Trp53tm2Tyj Commd10Tg(Vav1-icre)A2Kio (referred to as Trp53R172H/+) CD45.1, 5-6 weeks old, male and female (for chimera experiments with poly(I:C) and LPS challenge)
- C57/BL6 Trp53tm2Tyj Tg(Tal1-cre/ERT)42-056Jrg (referred to as Trp53LSL-R172H/+) CD45.2, 8-13 weeks old, males (for chimera experiments with poly(I:C) challenge)

Wild animals

This study did not involve wild animals.

Reporting on sex

- NOD.CB17-Prkdcscid IL2rgtm1/Bcgen mice (B-NDG, Envigo) : female
- C57/BL6 wild type mice CD45.1 or CD45.2 : male and female
- C57/BL6 Trp53tm2Tyj Commd10Tg(Vav1-icre)A2Kio (referred to as Trp53R172H/+) CD45.1 : male and female
- C57/BL6 Trp53tm2Tyj Tg(Tal1-cre/ERT)42-056Jrg (referred to as Trp53LSL-R172H/+) CD45.2 : males

Field-collected samples

Study did not involve field-collected samples.

Ethics oversight

Mouse experiments were approved by the French National Ethical Committee on Animal Care (n° 2020-007-23589) and by the UK University of Oxford Animal Welfare and Ethical Review Body (Project License P2FF90EE8).

Note that full information on the approval of the study protocol must also be provided in the manuscript.

Flow Cytometry

Plots

Confirm that:

☒ The axis labels state the marker and fluorochrome used (e.g. CD4-FITC).

☒ The axis scales are clearly visible. Include numbers along axes only for bottom left plot of group (a 'group' is an analysis of identical markers).

☒ All plots are contour plots with outliers or pseudocolor plots.

☒ A numerical value for number of cells or percentage (with statistics) is provided.

Methodology

Sample preparation	Human or mouse samples were stained in IMDM + 10% FCS or PBS + 5% FCS (respectively) with several antibodies, incubated during 20min at RT and washed before being analyzed. Staining for apoptosis was done in Annexin V binding buffer 1X (BD Biosciences). Cell cycle was assessed after fixation and permeabilization (BD Cytofix/Cytoperm and Permeabilization Buffer Plus, BD Biosciences).<br><br>All methods for sample preparation are fully described in the methods section of the manuscript.
Instrument	Cells were analyzed on a FACSCanto II or a BD Fortessa X20 (BD Biosciences) instrument.<br>Cells were sorted on a Influx Cell sorter (BD Biosciences), a BD Fusion I or Fusion II instruments (Becton Dickinson) or SH800S or MA900 sorters (SONY).
Software	Analysis of the flow cytometry data was performed using Kaluza (Beckman Coulter) or FlowJo (version 10.1, BD Biosciences) softwares.
Cell population abundance	Human and mouse haematopoietic stem and progenitor (HSPC) populations represent minor cell types (in the majority of cases, less than 1-5% of the total sample), except when they display a competitive advantage in the context of leukemic transformation. Sorting was performed in purity mode for bulk experiments and single-cell mode for single-cell sorting experiments. Post-sort purify was checked by sorting 100 cells from selected HSPC fractions (e.g. Lin-CD34+CD38- cells for human experiments and Lin-Sca1+ckit+ for mouse experiments) into an eppendorf tube containing 100 uL sorting buffer and analyzing the number of cells included within the same immunophenotype. Post-sort purity was consistently above 95%.
Gating strategy	Gating strategies are outlined in Extended Data Fig.2a (human HSPCs), Fig.5a (human cells from PDX models) and Fig.10b-c, l-n (mouse bone marrow and peripheral blood).

☒ Tick this box to confirm that a figure exemplifying the gating strategy is provided in the Supplementary Information.
